# A novel pathogenic missense *ADAMTS17* variant that impairs secretion causes Weill-Marchesani Syndrome with variably dysmorphic hand features

**DOI:** 10.1038/s41598-020-66978-8

**Published:** 2020-07-02

**Authors:** Daniel R. Evans, Jane S. Green, Somayyeh Fahiminiya, Jacek Majewski, Bridget A. Fernandez, Matthew A. Deardorff, Gordon J. Johnson, James H. Whelan, Dirk Hubmacher, Suneel S. Apte, Care4Rare Canada Consortium, Kym Boycott, Dennis Bulman, David Dyment, Alex McKenzie, Michael Brudno, Michael O. Woods

**Affiliations:** 1 Discipline of Genetics, Memorial University of Newfoundland, Faculty of Medicine, St. Johns, NL A1B 3V6 Canada; 2 Department of Human Genetics, McGill University, Montréal, QC H3A 1B1 Canada; 3 McGill University and Genome Québec Innovation Centre, Montréal, QC H3A 0G1 Canada; 4 Division of Genetics, Children’s Hospital of Philadelphia, Department of Pediatrics, University of Pennsylvania, Perelman School of Medicine, Philadelphia, Pennsylvania, PA 19104 USA; 5 Care of Dr. Jane Green, Discipline of Genetics, Memorial University of Newfoundland, Faculty of Medicine, St. Johns, NL A1B 3V6 Canada; 6 Memorial University of Newfoundland, Faculty of Medicine, Discipline of Surgery (Ophthalmology), St. Johns, NL A1B 3V6 Canada; 7 Orthopaedic Research Laboratories, Leni and Peter W. May Department of Orthopaedics, Icahn School of Medicine at Mount Sinai, New York, NY 10029 USA; 8 Department of Biomedical Engineering, Cleveland Clinic Lerner Research Institute, Cleveland, OH 44195 USA; 9 Children’s Hospital of Eastern Ontario Research Institute, University of Ottawa, Ottawa, ON K1H8L1 Canada; 10 Department of Medical Genetics, Faculty of Medicine & Dentistry, University of Alberta, Edmonton, AB T6G 2R3 Canada; 11 The Centre for Computational Medicine, The Hospital for Sick Children Research Institute, Toronto, ON M5G 1X8 Canada

**Keywords:** Medical genetics, Hereditary eye disease, Genetics research

## Abstract

Weill-Marchesani syndrome (WMS) is a rare disorder displaying short stature, brachydactyly and joint stiffness, and ocular features including microspherophakia and ectopia lentis. Brachydactyly and joint stiffness appear less commonly in patients with WMS4 caused by pathogenic *ADAMTS17* variants. Here, we investigated a large family with WMS from Newfoundland, Canada. These patients displayed core WMS features, but with proportionate hands that were clinically equivocal for brachydactyly. Whole exome sequencing and autozygosity mapping unveiled a novel pathogenic missense *ADAMTS17* variant (c.3068 G > A, p.C1023Y). Sanger sequencing demonstrated variant co-segregation with WMS, and absence in 150 population matched controls. Given *ADAMTS17* involvement, we performed deep phenotyping of the patients’ hands. Anthropometrics applied to hand roentgenograms showed that metacarpophalangeal measurements of affected patients were smaller than expected for their age and sex, and when compared to their unaffected sibling. Furthermore, we found a possible sub-clinical phenotype involving markedly shortened metacarpophalangeal bones with intrafamilial variability. Transfection of the variant ADAMTS17 into HEK293T cells revealed significantly reduced secretion into the extracellular medium compared to wild-type. This work expands understanding of the molecular pathogenesis of *ADAMTS17*, clarifies the variable hand phenotype, and underscores a role for anthropometrics in characterizing sub-clinical brachydactyly in these patients.

## Introduction

Weill-Marchesani syndrome (WMS) is a rare heritable connective tissue disorder (prevalence 1 in 100,000)^1^ that manifests with short stature, brachydactyly, thick skin, and joint stiffness^2^. Patients may also have microspherophakia, severe myopia, ectopia lentis, glaucoma and cataracts. Occasionally, there is pulmonary or aortic valve stenosis. WMS is caused by highly penetrant pathogenic variants in fibrillin-1 (*FBN1*), that cause autosomal dominant WMS (WMS2, OMIM #608328), while pathogenic variants in three other genes, *ADAMTS10* (WMS1, OMIM #277600), *LTBP2* (WMS3, OMIM#614819) *ADAMTS17* (WMS4, OMIM#613195) cause autosomal recessive WMS^3–5^. In addition to genetic heterogeneity, WMS also demonstrates subtle clinical heterogeneity, that together with its mechanistic basis, needs further clarification.

Fibrillin-1 forms tissue microfibrils that are key structural components of the extracellular matrix, also regulating TGFβ signaling and the biogenesis and homeostasis of elastic fibers, which support skin, ligaments, and blood vessels. Likewise, fibrillin microfibrils are the core structures of the ciliary zonule, which are compromised in WMS, and cause lens dislocations^6,7^. The other genes mutated in WMS encode ECM proteins that either localize to fibrillin-1 microfibrils in the ECM or can be involved in the formation of microfibrils. *LTBP2* encodes latent TGFβ binding protein-2, which directly binds fibrillin-1, and is required for stable assembly of microfibril bundles within the ciliary zonule^8,9^. *ADAMTS10* and *ADAMTS17* encode secreted metalloproteases, which require fibrillin-1 microfibrils for ECM localization and regulate assembly of microfibrils. *ADAMTS10* accelerates microfibril assembly through direct interactions with fibrillin-1^10^, whereas *ADAMTS17* binds fibrillin-1^11^, and may instead play a role in biogenesis and maturation of microfibrils, or act as a chaperone that facilitates fibrillin-1 secretion^12^.

Family studies that characterize WMS patients are essential toward advancing our understanding of these molecular pathways within the extracellular matrix. Pathogenic *ADAMTS17* variants were first described in 2009^5^. Review of the literature indicates there are eight pathogenic *ADAMTS17* variants reported in six studies, which collectively describe 18 WMS4 patients (Supplementary Table S1). Interestingly, among these reports, brachydactyly appears to be an uncommon manifestation. For example, Morales *et al*.^5^, and Khan *et al*.^13^, collectively identified four unrelated WMS families (i.e. nine affected patients) from Saudi Arabia. They reported four different pathogenic *ADAMTS17* variants (p.E820GfsX23; p.Q254X; c.1721 + 1 G > A; p.Asp218ThrfsX41) in these families. Notably, affected individuals displayed core WMS features, but consistently had normal (i.e. proportionate) hands, without any evidence of brachydactyly or joint stiffness. This observation led the authors to hypothesize that pathogenic *ADAMTS17* variants manifest as a ‘WMS-like’ syndrome (WMS4, OMIM #613195) typified by the above.

Subsequently, Radner *et al*.^14^ studied three families (i.e. four affected patients) with a complex phenotype involving both congenital ichthyosis and autosomal recessive WMS. They identified a contiguous 106.96 kb deletion involving *CERS3* and *ADAMTS17* (among other loci), in this unique 15q26.3 deletion syndrome. Notably, they described brachydactyly in three patients, and found joint stiffness in two patients. Unfortunately, given this causative deletion included multiple genes, it is difficult to correlate specific findings to *ADAMTS17*. Meanwhile, brachydactyly without joint stiffness was described by Shah *et al*.^15^, in the hands of one WMS patient with a pathogenic *ADAMTS17* splice variant (c.873 + 1 G > T). Subsequently, Yi *et al*.^16^, studied three WMS siblings with brachydactyly and identified a nonsense variant (p.Lys351*) in *ADAMTS17*. Finally, Karoulias *et al*.^12^, reported a pathogenic *ADAMTS17* missense variant (p.Thr343Ala) in a singleton WMS patient with no evidence of brachydactyly. There are inconsistencies however with how brachydactyly was ascertained among these studies (Supplementary Table S1). Diagnosis of brachydactyly can be either clinical, anthropometric or radiological; however, only one study defined their criteria for brachydactyly, and many appear to be based on clinical observation alone. Thus, to establish whether there is any utility in describing a ‘WMS-like’ syndrome, or more recently a specific designation of WMS-4 (OMIM #613195) specifically caused by *ADAMTS17* mutations, a more detailed analysis of the hand phenotype in patients with *ADAMTS17* variants is warranted.

The primary objective of our study was to solve the genetic etiology in a large multiplex family from Newfoundland, Canada with five affected siblings that displays autosomal recessive WMS. The family were first described in 1982, with core WMS features, but had broad appearing, proportionate hands, with stubby fingers, which were clinically equivocal for brachydactyly^17^. Subsequently, they were followed over a 40-year interval, during which no causative variant was found, prompting their enrollment in the FORGE Canada Consortium^18,19^, a large project designed to identify causative genetic variants in families with unexplained rare disease. Here, we report successful identification of a novel pathogenic *ADAMTS17* variant in this family with WMS. We performed deep phenotyping of patients’ hands using anthropometry to refine hand analysis and identified a possible sub-clinical phenotype. Functional characterization of the impact of this variant on secretion of ADAMTS17 into the extracellular medium, provides further insights into the mechanistic impact of the variant and pathophysiology of this disorder.

## Materials and Methods

### Patient Ascertainment

Family members live in a small rural community on the island of Newfoundland, Canada. Patients were first seen at an ophthalmology clinic by GJJ and were the subject of a case report in 1983^17^. Subsequently, they were referred to the Provincial Medical Genetics Program. Genetic testing during routine clinic visits did not identify any mutations in WMS genes known at the time (*ADAMTS10* & *FBN1)*. In 2007, family members gave written and informed consent to participate in an ocular genetics study. They consented to participate in the FORGE Canada Consortium study (now Care4Rare Canada Consortium) in 2012^18,19^. The current study was approved by the Health Research Ethics Board of Newfoundland & Labrador (HREB#2011.060) and all experiments were performed in accordance with relevant guidelines and regulations.

### Whole exome sequencing, variant filtering and homozygosity mapping

**Figure 1:**
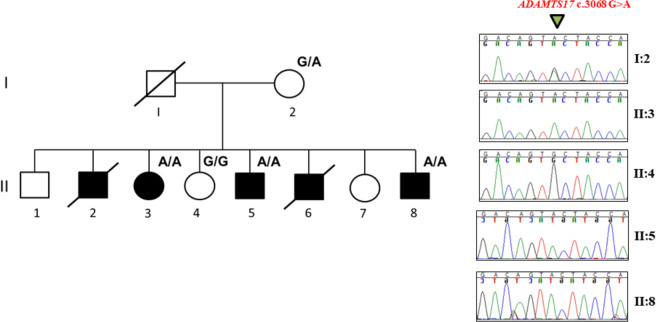
Large multiplex family with WMS with co-segregation of a novel pathogenic homozygous *ADAMTS17* missense variant (c. 3068 G > A; p. C1023Y).

Whole blood was drawn and genomic DNA was extracted from peripheral leukocytes by standard protocols. Whole exome sequencing (Illumina Hiseq. 2000) was performed according to protocols at the Genome Quebec Innovation Center, Montreal, Canada^18,19^. Briefly, 3 µg of genomic DNA from each affected individual (Fig. 1: II-5, II-3) was used for library preparation, and exome capture and enrichment was accomplished using the SureSelect Human Exome Kit V.4 (Agilent Technologies, Inc., Santa Clara, CA). Alignment, variant calling, and annotation were performed as per previous FORGE projects^20,21^. Mapping of high quality paired-end reads (100 bp) against the UCSC Genome hg19 was performed using BWA (v. 0.5.9)^22^. The Genome Analysis Toolkit (GATK)^23^ was used for local realignment around indels (short insertions and deletions), and for coverage assessment. Samtools (v. 0.1.17) mpileup^24^ and ANNOVAR^25^ were then used for variant calling and annotation, respectively. A custom filtering protocol was applied to the whole exome data and sequence variants were excluded in the event that: (1) <3 reads supported the alternative variant; (2) variant allele frequency was >0.05 in ExAC database and if variants were seen in >30 individuals in our database (~1000 exomes sequenced from provincial residents previously in our center); (3) variants were homozygous in ExAC database; (4) variants were synonymous; (5) variants were within 5’ UTRs or intronic variants outside of splice-site boundaries.

Finally, hypothesizing autozygosity for our family, mapping was performed using in-house scripts written in Perl, to identify regions of homozygosity (ROH). An ROH was defined as a span of at least 25 SNPs where no more than two are called as heterozygous and the remainder were homozygous.

### Validation of candidate variant, segregation analysis and population controls

To validate the candidate *ADAMTS17* variant, primers were designed using Primer3^26^, capturing exon 21 (forward primer: 5-′TCCCTTGACCTCA-3′; reverse primer: 5′-CACCGTCAGGGAG-3′). PCR conditions are available upon request. Sanger sequencing of PCR products was accomplished using an ABI 3130XL Genetic Analyzer, and chromatograms were interpreted using Sequencher 5.2.3.

In this manner, familial segregation was then tested in individuals I-2, II-3, II-4, II-5 and II-8 (Fig. 1). Finally, a cohort of 150 geographically matched population controls, from a previous study^27^ were screened for the *ADAMTS17* variant.

### Analysis of metacarpophalangeal lengths

Bilateral hand roentgenograms of an unaffected sibling (II-4) and two affected siblings (II-3, II-5) were obtained in 2016. To examine for dysmorphia, measurements of metacarpal bones, and proximal, middle and distal phalanges were taken. These measurements were compared against their expected proportions^28^, taking each person’s age and sex into account. In this study, we defined brachydactyly as hands in affected patients that were appreciably smaller compared to unaffected siblings.

### Cloning

Mammalian expression plasmids for full-length human ADAMTS17 and its proteolytically inactive mutant ADAMTS17-EA were described previously^11^. ADAMTS17-EA was used for initial analysis of the effect of the mutation on secretion, since the wild-type ADAMTS17 is autocatalytically processed and therefore difficult to detect in conditioned medium. To introduce the variant of interest, a synthetic DNA fragment spanning nucleotides 2799–3352 of *ADAMTS17* (Ref.Seq. XM_017021975) (Integrated DNA Technologies, Coralville, IA) was ligated with the gel-purified 8335 bp *Srf*I x *Afe*I fragment obtained from the ADAMTS17-EAplasmid, using the NEBuilder® HiFi DNA Assembly Kit (New England Biolabs, Ipswich, MA). Positive clones were screened via an additional *Sca*I restriction site introduced by the mutation and verified by DNA sequencing (Cleveland Clinic Lerner Research Institute Genomics Core).

### Cell culture, transfection, and collection of cell lysate and conditioned medium

Human embryonic kidney cells (HEK293T, ATCC, Manassas, VA) which were maintained in DMEM supplemented with 10% FBS, 100 units/ml penicillin and 100 μg/ml streptomycin in a 5% CO_2_ atmosphere in a humidified incubator at 37 °C. HEK293T cells were seeded in 12-well plates (BD Bioscience, San Jose, CA) and transfected in triplicate with 1 μg plasmid DNA/well using Lipofectamine 3000 according to the manufacturer’s protocol. 16–24 h after transfection, the medium was removed and the cells were rinsed with 1 ml PBS (137 mM NaCl, 2.7 mM KCl, 10 mM Na_2_KPO_4_, 1.8 mM KH_2_PO_4_) and incubated with 600 μl serum-free DMEM for 48 h. Conditioned medium was collected and cell debris were removed by centrifugation. The cell layer was rinsed with 1 ml PBS and cells were lysed in 0.1% NP40, 0.01% SDS, 0.05% Na-deoxycholate in PBS. Cell lysates were incubated for 5 min with rotation end-over-end and cleared by centrifugation (5 min, > 20,000 g, 4 °C).

### Western blotting

Equal volumes of conditioned medium or cell lysate from each transfection were subjected to 7.5% SDS-PAGE under reducing conditions. Proteins were transferred onto PVDF membranes (Immobilon F, EMD Millipore, Billerica, MA) for 1.5 h at 70 V at 4 °C in 25 mM Tris, 192 mM glycine, 20% methanol buffer. The membrane was blocked with 5% (w/v) milk in TBS (10 mM Tris-HCl, pH 7.2, 150 mM NaCl) for 1 h at room temperature and anti-myc (clone 9E10, Invitrogen, 1:500) was diluted in 5% (w/v) milk in TBS + 0.1% Tween 20 (TBST) and incubated overnight at 4 °C. Membranes were washed three times with TBST for 5 min at RT and incubated with fluorophore-labeled IRDye goat-anti-mouse secondary antibody (1:10,000) (LI-COR Biosciences, Lincoln, NE) in 5% milk in TBST + 0.01% SDS for 1 h at room temperature. Membranes were washed three times with TBST for 5 min, once with TBS for 5 min at room temperature, and scanned wet on an Odyssey CLx scanner (LI-COR Biosciences, Lincoln, NE). The fluorescent signal was quantified using the Image Studio software.

## Results

Five out of eight siblings were affected with WMS in this family (Fig. 1). The pattern of WMS is autosomal recessive, and both parents live in a small rural community, sharing the same surname prior to marriage, but are unaware of any consanguinity. Their 40-year clinical histories are summarized in Table 1. Affected individuals have characteristic WMS features such as short stature (mean height 156.5 cm), early onset myopia (5/5 siblings), microspherophakia (4/5 siblings) and ectopia lentis (4/5 siblings). They all had high IOPs diagnosed from early ages (10–26 years). Cataracts and glaucoma developed in 2/5. Longitudinal clinical histories for each patient are provided (Supplementary Results). Joint stiffness was an uncommon manifestation and only 1/5 siblings (sibling II-8) presented with limited range of motion in the wrists and shoulders and had a winged scapula.

**Table 1:** Clinical History of Five WMS Patients with Autosomal Recessive Inheritance.

Clinical Features	II-2	II-3	II-5	II-6	II-8
Sex	M (30)	F (68)	M (64)	M (43)	M (55)
Myopia	Y (13)	Y (10)	Y (16)	Y (14)	Y (10)
Microspherophakia	Y (13)	Y (10)	Y (18)	Y (14)	Spherophakia (10)
Ectopia Lentis	Y [OU] (13)	Y [OU] (10)	Y (OS) (43)	Y (14)	N
Elevated IOP	Y (13)	Y (10)	Y (26)	Y (22)	Y (10)
Glaucoma	N	Y (OU) (28)	Y (25)	N	N
Cataracts	N	Y (OU; 50, 51)	Y (OD; 2), (OS;49)	N	N
Other Ocular History	N	IOLs, retinal tear and detachme8nt (OD; 48),	Penetrating trauma (OD 2)	N	N
Adult Height	160 cm	149 cm	157 cm	161 cm	155.5 (mid-late teens)
Clinical Examination of Hand	NA	Wide and stubby hands with bil 5^th^ finger clinodactyly	Broad hands and feet. Fingers and toes short and wide	Short and spade-like hands with stubby fingers. Prominent knuckles and knobby interphalangeal joints.	NA
Roentgenogram	N	Unilateral shortening (R) 1^st^ proximal phalanx (−5.3 SD)	Unilateral shortening (L) 1^st^ metacarpal (−7 SD)	Bil. shortening 4th & 5th metacarpals (1982)	Bil. shortening of 1^st^, 4^th^, 5^th^ metacarpals (1982)
Joint Stiffness	N	N (~30 s)	N	Limited ROM wrists & shoulders	N
Cardiac Anomalies	NA	NA	N	N	Queried pulmonary stenosis with early I/IV systolic murmur that eventually resolved with later normal echocardiogram (17)
Treatments	N	Timolol, OU PI (28), OS lensectomy, pars plana vitrectomy and lens replacement with an IOL (58), timoptic and truspot BID for glaucoma	Timolol (25), L peripheral iridectomy (26), IOL (49)	OU PI (22)	Timolol ineffective, OU peripheral iridectomy (17)
Systemic Features	N	Endometriosis 1978–1979, (L) ovarian cystectomy (1979), total hysterectomy (1980), Bil salpingectomy with left oophorectomy	CRC & polyps (53)	Winged scapula	Cognitive impairment
Status	D. (accidental) (30)	L	L	D. (unknown) (43)	L

Abbreviations: OU (both eyes), OS (left eye), OD (right eye), IOL (intraocular lens), PI (peripheral iridectomy), bil (bilateral), ROM (range of motion), SD (standard deviations), BID (twice daily), CRC (colorectal cancer), Y (yes), N (no), NA (not available), L (left), R (right), D. (deceased).

**Figure 2:**
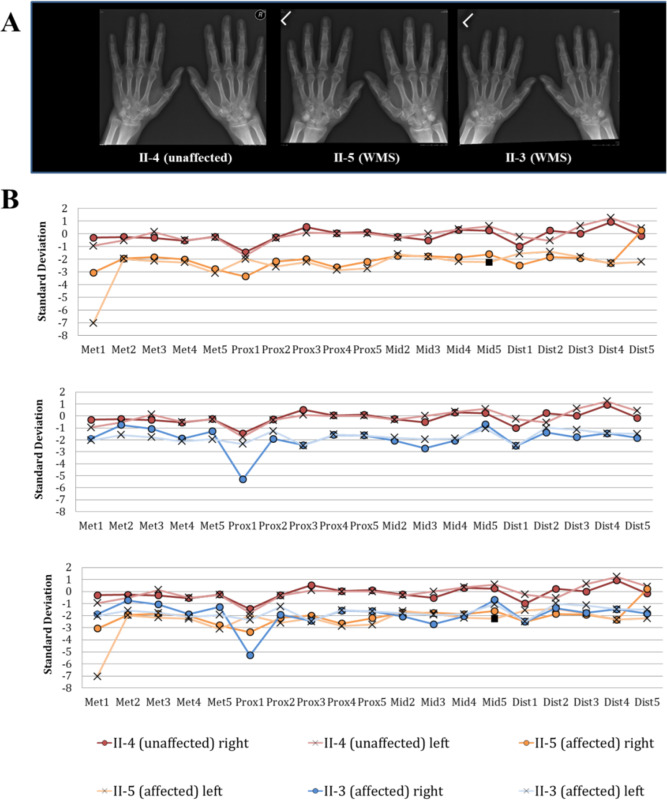
Roentgenogram and anthropometric analysis of family members demonstrates intrafamilial variability of hand features. Bilateral roentgenograms (**A**) of unaffected sibling II-4 (left) compared with affected sibling II-5 (center) and II-3 (right). There is bilateral 5^th^ finger clinodactyly in II-3 and, overall, the hands of affected siblings are shorter and stubbier than their unaffected sibling. Row B shows age and sex corrected anthropometrics of metacarpophalangeal bones. Unaffected sibling II-4 is compared against affected sibling II-5 (top panel), and II-3 (middle panel), or both siblings (bottom panel). There was marked shortening (−7 S.D) of left 1^st^ metacarpal in II-5 (light orange line) and shortened (−5.3 S.D) right 1^st^ proximal phalanx in II-3 (dark blue line). Overall, measurements of the affected siblings (orange and blue lines) are shorter (−1 to 3 S.D) as compared to their unaffected sibling (1 to −1 S.D; red lines).

We next analyzed the hand phenotype in these patients, given the incomplete clinical penetrance of brachydactyly in previous descriptions of individuals with WMS4. The hands of our patients appeared wide with stubby fingers, but clinically, were proportionate and equivocal for brachydactyly^17^. Roentgenograms demonstrated shortened metacarpophalangeal bones. For example, sibling II-6 had bilateral shortening of 4^th^ and 5^th^ metacarpals, while sibling II-8 had bilateral shortening of 1^st^, 4^th^ and 5^th^ metacarpals (Table 1). To perform a deeper phenotyping, new roentgenograms were obtained from available affected siblings (II-3, II-5) and their unaffected sibling (II-4) (Fig. 2). Anthropometrics of the hands, adjusted for age and sex, were applied to characterize differences in metacarpophalangeal measurements^28^ (Fig. 2B). In the unaffected sibling (II-4), metacarpophalangeal measurements predominantly varied between 1 and −1 standard deviations from expected values. However, in the affected siblings (II-3 and II-5), these values predominantly varied from −1 to −3 standard deviations below their expected values. Moreover, in affected sibling II-5 there was marked unilateral shortening of the 1^st^ metacarpal on the left hand (−7 standard deviations below expected). Likewise, we observed a unilaterally shortened 1^st^ proximal phalanx (−5.3 standard deviations below expected) on the right hand of affected sibling II-3.

**Figure 3:**
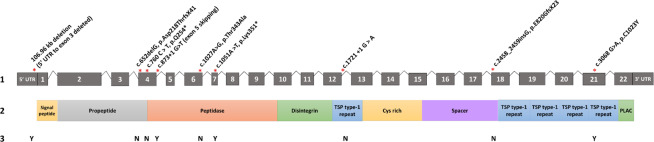
Distribution of reported pathogenic *ADAMTS17* variants in WMS families worldwide. 1. Exon structure of *ADAMTS17*. Asterisks (red) show approximate position of reported pathogenic variants. Introns are not drawn to scale. 2. Shows the relative position of protein domains coinciding with exonic structure. 3. Describes whether a study reported brachydactyly (Y) or proportionate hands (N).

Genetic analysis was pursued as part of the FORGE Canada Consortium. Whole exome sequencing and homozygosity mapping of genomic DNA samples from siblings II-3 and II-5 revealed a homozygous missense variant in *ADAMTS17* (NM_139057.3: c.3068 G > A: p.C1023Y). GnomAD reports that p.C1023Y is rare, with a minor allele frequency (MAF) of 0.0016% (3 out of 187,624 alleles; no homozygotes). Sanger sequencing for p.C1023Y in 150 population matched controls from Newfoundland was negative. Familial co-segregation analysis corroborated the expected pattern of transmission for a fully penetrant, autosomal recessive disorder (Fig. 1). Several *in silico* tools predicted highly deleterious consequences for this substitution. For example, SIFT predicted deleterious effects (score = 0), PolyPhen-2 predicted damaging (score = 1.0), MutationTaster predicted disease-causing (score: 0.99), CADD score was 32, and GERP score was 5.23, suggesting a region of high evolutionary conservation. Positionally, p.C1023Y substitution resides in a thrombospondin type 1 repeat, which spans amino acids 976–1028 of the ADAMTS17 polypeptide (Fig. 3).

**Figure 4:**
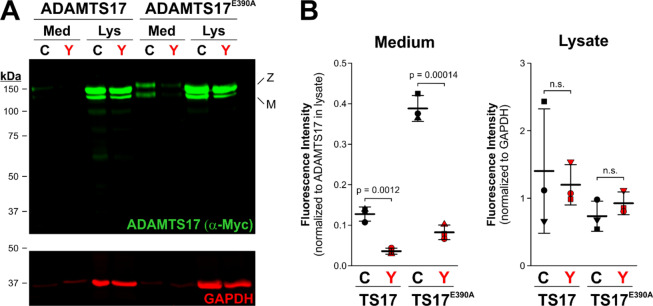
Impaired secretion of the *ADAMTS17* p.C1023Y mutant in transfected HEK293T cells. (**A**) Western blot comparing levels of mutant and wild-type ADAMTS17 (detected by anti-myc, green, upper panel) in the medium and lysate. There is a noticeable reduction in intensities in the medium of p.C1023Y expressing cells (“Y” allele in red) compared to wild type (“C” allele) in both ADAMTS17 and ADAMTS17-E^390^A constructs. No difference in signal intensity was seen in the corresponding cell lysates. Z, zymogen; M. mature enzyme. The lower panel shows the corresponding GAPDH western blot (red). (**B**) Quantification of ADAMTS17 fluorescence intensity in the medium normalized to ADAMTS17 in the corresponding lysate. Note the reduced intensities for ADAMTS17 levels in the medium in the p.C1023Y mutants (“Y” allele in red) (p = 0.0012 ADAMTS17 and p = 0.00014 in ADAMTS17^E390A^).

Thus, having identified a novel pathogenic *ADAMTS17* variant, we proceeded to characterize the functional consequences of p.C1023Y (Fig. 4). HEK293T cells were transfected with a plasmid where the p.C1023Y variant was introduced in ADAMTS17 and its active site mutant form (p.E390A) to analyze secretion of ADAMTS17 into the extracellular medium. Fluorescence intensities of ADAMTS17 p.C1023Y mutants were compared against wild type constructs or active site mutant constructs in either extracellular medium or cell lysates. The p. E390A active site mutants are useful as they do not undergo autoproteolysis, and thus are more readily detected in the medium of transfected cells^11^. A significant reduction of ADAMTS17 p.C1023Y mutants was seen in the extracellular medium in both wild type (p = 0.0012) and p.E390A constructs (p = 0.00014). In cell lysates, there were no significant differences in wild type or p. E390A constructs harboring either wild type residue or the p.C1023Y substitution. Thus, ADAMTS17 expression was not significantly reduced inside HEK293T cells nor did it accumulate inside the cell, whereas secretion into extracellular medium was significantly impaired in p.C1023Y mutants.

## Discussion

Here, we investigated the molecular underpinnings for WMS in a large family from Newfoundland & Labrador and identified a novel homozygous pathogenic missense *ADAMTS17* variant (NM_139057.3: c.3068 G > A: p.C1023Y). Having been followed since 1983, this family provides us with a unique and longitudinal view of their WMS history (Supplementary Results). Patients demonstrated core WMS features such as short stature, early onset myopia, microspherophakia and ectopia lentis, but visually their hands were not strongly suggestive of brachydactyly (Table 1). Interestingly, hand roentgenograms revealed shortened metacarpophalangeal bones with intrafamilial variability. Anthropometric analysis allowed us to delineate this sub-clinical hand phenotype in our WMS4 patients (Fig. 2). The analysis showed that hands of the affected siblings are objectively smaller than their unaffected sibling. For example, in the unaffected sibling (II-4), metacarpophalangeal measurements predominantly ranged from 1.0 to −1.0 standard deviations around expected values. This contrasted with the affected siblings (II-3, II-5), whose metacarpophalangeal measurements predominantly ranged between −1.0 and −3.0 standard deviations below expected values. Therefore, siblings II-3 and II-5 have a mild brachydactyly. Notably, our deep phenotyping in II-3 and II-5 also quantified a marked unilateral shortening of metacarpophalangeal bones (i.e. 1^st^ proximal phalanx and 1^st^ metacarpal, respectively) in these two family members. Thus, our findings further underscore the clinical heterogeneity and intrafamilial variability of the brachydactyly phenotype in WMS and emphasize that the overall clinical hand appearance can mask significant anomalies in individual metacarpals, which can be elicited by antropometry.

An interesting correlation with published literature is that brachydactyly is uncommonly found among patients with pathogenic *ADAMTS17* variants. For example, after excluding the 15q26.3 deletion syndrome, 10 out of 14 WMS patients with reported pathogenic variants had reportedly normal hands (Supplementary Table S1). However, many of these studies relied solely on a clinical approach to reporting brachydactyly. In our study, WMS patients had hands that were proportionate and clinically equivocal, but roentgenograms revealed variably dysmorphic features, which we quantified with anthropometrics (Fig. 2). Our findings suggest the possibility of a sub-clinical phenotype, which may have been overlooked among previously reported studies. We therefore emphasize the importance of applying roentgenogram and anthropometric analysis when addressing the question of the *ADAMTS17* hand phenotype.

Ultimately, it remains plausible that the hand phenotype in *ADAMTS17* mutations demonstrates a spectrum of WMS severity. For example, some patients will develop normal hands, others might have sub-clinical dysmorphia, while others display more obvious brachydactyly. Future studies might consider whether a unique function of ADAMTS17 within the extracellular matrix, or potential functional overlap with ADAMTS10 leading to a degree of compensation might explain the variability in the hand phenotype compared to the other causative genes; since ADAMTS10 is universally expressed in cells in mouse paws^29^. Present evidence does not suggest any genotype-phenotype correlation when comparing the position of *ADAMTS17* variants and the presence of brachydactyly (Fig. 3), recognizing the limitations given the low number of reported cases. Importantly, the p.C1023Y variant had a severe impact on the secretion of ADAMTS17, meaning that it cannot inform about a possible domain-specific effect for genotype-phenotype correlations.

Currently, there are nine pathogenic variants in *ADAMTS17* that cause WMS4. There are two frameshift mutations (p.E820GfsX23; p.Asp218ThrfsX41), two splice site mutations (c.1721 + 1 G > A; c.873 + 1 G > T), two nonsense mutations (p.Q254*; p.Lys351*), a contiguous gene deletion on 15q26.3, and now two missense variants (p.Thr343Ala; p.C1023Y), which occur in multiple regions of *ADAMTS17* (Supplementary Table S1, Fig. 3). Most previous studies show altered mRNA isoforms caused by high impact mutations with subsequent predictions of truncated ADAMTS17 proteins. Studies of a missense *ADAMTS17* variants (p.Thr343Ala; p.C1023Y) by Karoulias *et al*.^12^, and our current study respectively, further delineate the functional consequences of pathogenic *ADAMTS17* variants. The p.Thr343Ala variant lies in a catalytic domain and results in decreased secretion of ADAMTS17 into the extracellular matrix, whereas the p.C1023Y variant resides in the thrombospondin-1 repeat domain of ADAMTS17 and results in comparable deficiency in secretion. Our analysis of ADAMTS17 p.C1023Y mutants in HEK293T cells revealed significantly reduced levels of ADAMTS17 in cellular medium as compared with wild-type ADAMTS17 (p = 0.0012) and ADAMTS17^E390A^ (p = 0.00014) constructs (Fig. 4). Together, these studies of the *ADAMTS17* missense variants demonstrate that ADAMTS17 secretion can be reduced or eliminated as a consequence of missense mutations, suggesting that the consequences of these pathogenic variants is a functional loss of ADAMTS17 in the extracellular matrix or at the cell surface. Their effect is thus independent of the location of the mutations in ADAMTS17 protein, and is unlikely to reflect impairment of domain-specific interactions or functions.

In conclusion, this study of a large multiplex family with WMS identified a novel pathogenic missense variant (p.C1023Y) in *ADAMTS17*. This variant caused severely reduced secretion of ADAMTS17 into the extracellular medium, suggesting functional loss of ADAMTS17 rather than impairment of a domain-specific function or interaction. Deep phenotyping of our patients’ hands provides novel observations regarding the spectrum of the WMS hand phenotype, highlighting a possible sub-clinical phenotype with intrafamilial variability. This work expands our understanding of the molecular pathogenesis of *ADAMTS17*, clarifies the resultant hand phenotype, and underscores a role for anthropometrics in characterizing brachydactyly in these patients.

## Supplementary information

Supplementary Information.
