# Supplementary material for: A novel pathogenic missense ADAMTS17 variant that impairs secretion causes Weill-Marchesani Syndrome with variably dysmorphic hand features
Source: Sci Rep. 2020 Jul 2;10:10827. doi: 10.1038/s41598-020-66978-8 (PMC7331723; doi:10.1038/s41598-020-66978-8)
Supplement: Supplementary file 1 — Supplementary Information. [file 41598_2020_66978_MOESM1_ESM.pdf]

A novel pathogenic missense *ADAMTS17* variant that impairs secretion causes Weill-Marchesani Syndrome with variably dysmorphic hand features

Daniel R. Evans[1], Jane S. Green[1], Somayyeh Fahiminiya[2,3], Jacek Majewski[2,3], Bridget A. Fernandez[1], Matthew A. Deardorff[4], Gordon J. Johnson[5], James H. Whelan[6], Dirk Hubmacher[7], Suneel S. Apte[8], Care4Rare Canada Consortium and Michael O. Woods[1]*

[1]Discipline of Genetics, Memorial University of Newfoundland, Faculty of Medicine, St. Johns, NL, A1B 3V6 Canada.
[2]Department of Human Genetics, McGill University, Montréal, QC, H3A 1B1 Canada.
[3]McGill University and Genome Québec Innovation Centre, Montréal, QC, H3A 0G1 Canada.
[4]Division of Genetics, Children's Hospital of Philadelphia, Department of Pediatrics, University of Pennsylvania, Perelman School of Medicine, Philadelphia, Pennsylvania, PA 19104 USA.
[5]Care of Dr. Jane Green, Discipline of Genetics, Memorial University of Newfoundland, Faculty of Medicine, St. Johns, NL, A1B 3V6, Canada.
[6]Memorial University of Newfoundland, Faculty of Medicine, Discipline of Surgery (Ophthalmology) St. Johns, NL, A1B 3V6 Canada.
[7]Orthopaedic Research Laboratories, Leni and Peter W. May Department of Orthopaedics, Icahn School of Medicine at Mount Sinai, New York, NY, 10029, USA.
[8]Department of Biomedical Engineering, Cleveland Clinic Lerner Research Institute, Cleveland, OH, 44195, USA.

Corresponding Author

Michael O. Woods
Associate Professor
Discipline of Genetics
Faculty of Medicine
Memorial University of Newfoundland
300 Prince, Phillip Drive, St. John's, NL, Canada
Phone: 709-351-2584
Email: mwoods@mun.ca

Authors	Family ID	# Affected	Mutation	Functional Studies	Brachydactyly	Joint Stiffness	Criteria for Brachydactyly
Morales et al., 2009	Family A	4	c.2458_2459insG (p.E820GfsX23)	mRNA analysis showing stable transcript expression, no in vitro mutation modelling	0/4	0/4	Clinical diagnosis. Brachydactyly defined as presence of short, stubby fingers in comparison to normal-appearing hands in unaffected family members
	Family F	2	c.760 C > T (p.Q254X)	mRNA analysis showing stable transcript expression, no in vitro mutation modelling	0/2	0/2	
	Family D	1	c.1721 +1 G > A	mRNA analysis showed 3 isoforms with frameshift induced by skipping of exon 12 or retained intron 12. No in vitro modelling.	0/1	0/1	
Khan et al., 2012	Study Family	2	c.652delG (p.Asp218ThrfsX41)	No	0/2	0/2	Not formally described. Diagnosis appears clinical
Radner et al., 2013	Founder mutation in 3 families (not enough data to differentiate between each family)	4	Homozygous contiguous 106.96 kb gene deletion syndrome of 15q26.3 (CER3, ADAMTS17)	No	3/4	2/4	Not formally described. Diagnosis appears clinical
Shah et al., 2014	Study Family	1	c.873+1 G>T	mRNA analysis showed exon 5 skipping, no in vitro modelling	1/1	0/1	Not formally described. Clinical diagnosis supported by hand roentgenogram
Yi et al., 2019	Study Family	3	c.1051A >T (p.Lys351*)	No	3/3	Not reported	Not formally described. Diagnosis appears clinical
Karoulias et al., 2019	Study Family	1	c.1027A > G (p.Thr343Ala)	Recombinant p.Thr343Ala mutants in HEK293T cells shows decreased secretion into the medium	0/1	0/0	Not formally described. Absence of brachydactyly supported by hand roentgenogram

Supplementary Table 1. Reported *ADAMTS17* pathogenic variants, predicted effects, and manifestation of brachydactyly or joint stiffness in affected WMS patients.

Supplementary Clinical Data

The oldest affected individual (Table 1: II-2) first presented with myopia at 13 years of age. He had microspherophakia and iridodonesis with lenses displaced nasally. Shallow anterior chambers were noted. Peripheral iridectomies were recommended for this individual, but he died accidentally at age 30 before any treatment took place. Other family members were examined. His younger sister (Table 1: II-3) had high myopia at age 10 with increasing intraocular pressure (IOP). She subsequently received bilateral peripheral iridectomies at age 28. She had a stocky habitus; her approximate adult height was 149 cm. She did not report any difficulties with range of motion in her fingers or wrists. Visually, her hands appeared wider and stubbier than normal, with a lateral curvature of both her fifth fingers at the distal interphalangeal joints. Like her older brother, she had microspherophakia with both lenses positioned only slightly nasally (subluxation). She had a right retinal tear and detachment repair (age 48), and cataract extractions (ages 50 and 51) with replacement by an intraocular lenses (IOL). At 58 years old, she had extraction of a dislocated left IOL, pars plana vitrectomy and lens replacement with an anterior chamber IOL. Her visual acuity returned to R: $20/25^{+}$ and L: $20/40^{-}$ following surgery. No cardiac abnormalities were reported. She did not report any new stiffness in her joints, when specifically asked about her ability to make a closed fist.

Her younger brother (Table 1: II-5) developed a traumatic cataract from a penetrating right eye injury at age 2. He had microspherophakia identified by age 18, and subsequently had a left peripheral iridectomy due to increasing IOP at age 26. In his late twenties, his build was stocky, with height of 157 cm. He had a normal echocardiogram and no cardiac abnormalities. His hands and feet were broad, and fingers and toes appeared

short and wide. He had a shortened first metacarpal on his left hand. There was no restriction of joint mobility. He had cataract extraction and replacement with an anterior chamber IOL (age 49). At age 53 he had colon cancer with several polyps, as did his mother.

The next sibling was a brother (Table 1: II-6), who had myopia and microspherophakia noted at age 14. Marked iridodonesis was observed, and due to increasing IOP, he had bilateral peripheral iridectomies at age 22. His approximate height was 161 cm in his early twenties. In 1982, hand X-rays demonstrated bilateral shortening of the fourth and fifth metacarpals. He died at age 43.

Finally, the youngest brother (Table 1: Individual II-8) was first examined at age 10. He had myopia with spherical lenses. As his bilateral IOPs increased, he was first treated with timolol, and then had bilateral peripheral iridectomies at age 17. He has cognitive impairment (thought to be from birth trauma) and had a heart murmur as a child. He was diagnosed with pulmonary stenosis by a cardiologist (grade I/VI) at this time. A subsequent echocardiogram performed in 1982 indicated the pulmonary stenosis was not clinically significant. Roentgenograms in 1982 demonstrated shortening of the first, fourth and fifth metacarpals in both hands. His height was 156 cm in his mid-to late teens. On physical examination, his hands were described as short and spade-like with stubby fingers, and prominent knuckles and knobby interphalangeal joints. He had markedly decreased limitation of movement in wrists and shoulders, and a winged scapula.
